# Pre-treatment preferences and characteristics among patients seeking in vitro fertilisation

**DOI:** 10.1186/1742-4755-6-21

**Published:** 2009-12-17

**Authors:** Anthony PH Walsh, Gary S Collins, Monique Le Du, David J Walsh, Eric Scott Sills

**Affiliations:** 1 The Sims Institute/Sims International Fertility Clinic, Dublin, Ireland; 2 Centre for Statistics in Medicine, University of Oxford, Oxford, UK; 3 Faculty of Nursing, Princess Grace Hospital Centre, Monte Carlo, Monaco

## Abstract

**Background:**

This study sought to describe patient features before beginning fertility treatment, and to ascertain their perceptions relative to risk of twin pregnancy outcomes associated with such therapy.

**Methods:**

Data on readiness for twin pregnancy outcome from in vitro fertilisation (IVF) was gathered from men and women before initiating fertility treatment by anonymous questionnaire.

**Results:**

A total of 206 women and 204 men were sampled. Mean (± SD) age for women and men being 35.5 ± 5 and 37.3 ± 7 yrs, respectively. At least one IVF cycle had been attempted by 27.2% of patients and 33.9% of this subgroup had initiated ≥3 cycles, reflecting an increase in previous failed cycles over five years. Good agreement was noted between husbands and wives with respect to readiness for twins from IVF (77% agreement; Cohen's K = 0.61; 95% CI 0.53 to 0.70).

**Conclusion:**

Most patients contemplating IVF already have ideas about particular outcomes even before treatment begins, and suggests that husbands & wives are in general agreement on their readiness for twin pregnancy from IVF. However, fertility patients now may represent a more refractory population and therefore carry a more guarded prognosis. Patient preferences identified before IVF remain important, but further studies comparing pre- and post-treatment perceptions are needed.

## Background

Throughout Europe, a steady increase in number of patents seeking infertility treatment has been noted over recent years. As more individuals seek in vitro fertilisation (IVF), the success rates associated with this treatment have improved and the medications required to achieve pregnancy have become more patient-friendly. Yet little is known about the medical and social features of this growing patient population, particularly in Ireland. In this report, patients about to embark on an IVF cycle in Ireland were surveyed with a view to describe demographic and clinical characteristics. This investigation also collected data about patient pre-treatment anxiety, especially about a twin pregnancy from their upcoming fertility cycle, and assessed variance between husbands and wives regarding this outcome after IVF.

## Methods

All new IVF consultations were prospectively identified and sampled with a voluntary, anonymous questionnaire collected prior to beginning treatment during a 45-d study period. The following parameters were queried: age, previous fertility treatment, family history of twins, availability of frozen embryos at baseline (*i.e*., from another clinic), duration of infertility, and readiness for/anxiety level regarding twin pregnancy outcome from IVF. Responses from husbands and wives were tabulated separately. Additionally, couples were asked about how certain IVF outcomes might influence their subsequent decisions regarding the fate of any possible cryopreserved embryos deriving from treatment. For analysis, all respondents were stratified into the following five age groups: <30, 30-34, 35-39, 40-44, and >44 yrs. Association between categorical outcomes was assessed by χ^2 ^test, and variability between husbands vs. wives was measured by Cohen's kappa for agreement. An adjusted bootstrap percentile 95% confidence interval for kappa was calculated based on 1000 samples.

## Results

Questionnaires were returned by 206 women and 204 men attending for new reproductive endocrinology consultation at Sims International Fertility Clinic in Dublin; clinical and demographic features in this group are presented in Table 1. During the study period a total of 227 new IVF patients were seen, yielding a response rate of 90.7% for this investigation. In our study population, mean (± SD) patient age was 35.5 ± 5 yrs for women and 37.3 ± 7 yrs for men. Mean duration of infertility was 2.7 yrs. In this group 56 (27.2%) had already completed at least one IVF cycle elsewhere. Patients having initiated ≥3 IVF cycles comprised 33.9% of the sample, consistent with prior data in this population reflecting an increased number of failed cycles over a five-year interval (3.5 vs. 4.5; *p *< 0.001). A pregnancy had been achieved by 79 study patients (38.3%) prior to initial consultation here. Patient baseline awareness of ovulation induction agents' association with an increased risk of multiple gestation was affirmed by 195 patients (94.7%). Analysis of reported data showed husbands and wives were in general agreement regarding their readiness for twins from IVF, as shown in Table 2 (77% agreement, Cohen's K = 0.61; 95% CI 0.53 to 0.70). Stratification of both partners by age did not reveal any associations with pre-treatment anxiety for a twin pregnancy outcome (Table 3). Concordance was noted in 91.3% of couples regarding the disposition of cryopreserved embryos regardless of whether IVF resulted in successful twin delivery (either sex not specified, same sex, or different sex twins), showing a tendency for both partners to be more likely to maintain embryos in storage. Uncertainty as to what they might do with any possible frozen embryos was indicated by 89 patients, and the option of placing embryos in an anonymous donation programme or making them available for research was selected less often (Table 4).

**Table 1:** Summary of demographic and clinical characteristics of patients seeking reproductive endocrinology consultation.

Parameter	Value
Mean age (SD) [range]	
Women (n = 206)	35.49 (4.6) [24 to 46]
Men (n = 204)	37.31 (5.6) [24 to 58]
Prior pregnancy together, n (%)	79 (38.3)
	
Total number of prior pregnancies together, n (%)	
1	40 (50.6)
2	19 (24.1)
3	13 (16.5)
≥ 4	5 (6.3)
Not stated	2 (2.5)
	
Infertility duration (yrs) - median (IQR)	2.7 (1, 4)
	
Prior IVF experience, n (%)	56 (27.2)
	
Number of prior IVF cycles, n (%)	
1	18 (32.1)
2	19 (33.9)
3	10 (17.9)
4	4 (7.1)
≥ 5	5 (8.9)
	
Prior IVFs resulting in a delivery, n (%)	11 (19.6)
	
Any frozen embryos in storage, any facility? n (%)	10 (4.9)
	
Prior IUI treatment, n (%)	30 (14.6)
Number of IUIs, median (range)	2 (1, 20)
	
Outcome of IUI, n (%)	
No pregnancy	27 (90)
Pregnancy	3 (10)
Any family history of twins or triplets? n (%)	78 (37.9)
	
Aware that fertility treatments (including IVF) are associated with an increased risk of multiple gestation? n (%)	195 (94.7)

*Notes*: IVF = in vitro fertilisation IUI = intrauterine insemination IQR = interquartile range

**Table 2:** Summary of data on readiness for twins as reported by husbands and wives before embarking on fertility treatment.

	Twins would be a very undesirable outcome	I would be a little worried about having twins	It is irrelevant & I don't care about twins	I'm only mildly interested in twins	I would be very excited - twins would be highly desirable	TOTAL
**Wife**	2 (1.0)	29 (14.6)	28 (13.6)	21 (11.2)	121 (59.7)	206
**Husband**	4 (2.0)	23 (11.4)	44 (21.9)	17 (8.5)	113 (56.2)	201

*Notes: *Not all patients answered every question. Cohen's Kappa for agreement, κ = 0.61 (95% CI 0.53 to 0.70) [1000 bootstrap simulations; BCa CI], showed good agreement between husband vs. wives on readiness for twins (77% agreement; κ = 0.61; 95% CI 0.53 to 0.70).

**Table 3:** Data sampled from couples on readiness for twins before beginning fertility treatment, stratified by age.

	Twins would be a very undesirable outcome	I would be a little worried about having twins	It is irrelevant & I don't care about twins	I'm only mildly interested in twins	I would be very excited - twins would be highly desirable	TOTAL
**Age (yrs)**						
**Wife**						
**<30**		1 (4.5)	3 (13.6)	3 (13.6)	15 (68.2)	22 (10.7)
**30-34**	1 (1.6)	7 (11.3)	11 (17.7)	7 (11.3)	36 (58.1)	62 (30.1)
**35-39**	1 (1.3)	17 (21.8)	9 (11.5)	9 (11.5)	42 (53.8)	78 (37.9)
**≥40**		5 (11.4)	5 (11.4)	4 (9.1)	30 (68.2)	44 (21.4)
**TOTAL**	2 (1.0)	30 (14.6)	28 (13.6)	23 (11.2)	123 (59.7)	206
**Husband**						
**<30**			3 (33.3)		6 (67.3)	9 (4.5)
**30-34**	1 (1.8)	3 (5.3)	16 (28.1)	5 (8.8)	32 (56.1)	57 (28.4)
**35-39**	2 (2.6)	13 (17.1)	11 (14.5)	9 (11.8)	41 (53.9)	76 (37.8)
**40-44**	1 (2.6)	5 (13.2)	9 (23.7)	1 (2.6)	22 (57.9)	38 (18.9)
**≥ 45**		2 (9.5)	5 (23.8)	2 (9.5)	12 (57.1)	21 (10.4)
**TOTAL**	4 (2.0)	23 (11.4)	44 (21.9)	17 (8.5)	113 (56.2)	201

*Note: *Neither maternal age (*p *= 0.718) nor paternal age (*p *= 0.656) was associated with anxiety about twins in an upcoming fertility treatment cycle.

**Table 4:** Patient response to possible embryo disposition status as a function of future pregnancy outcome from IVF.

Hypothetical family scenario	*n*
If treatment at our centre resulted in a successful delivery of your healthy baby, and you had extra embryos remaining in storage here, what do you think you would be most likely to do with them?	
Maintain them in storage, and plan for another attempt for pregnancy	127
Donate them for stem-cell research	3
Make them available for anonymous adoption by another couple	6
Undecided	65
Not answered	5
	
If treatment at our centre resulted in a successful twin delivery of two healthy babies (sex not specified) and you had extra embryos remaining in storage here, what do you think you would be most likely to do with them?	
Maintain them in storage, and plan for another attempt for pregnancy	96
Donate them for stem-cell research	6
Make them available for anonymous adoption by another couple	12
Undecided	87
Not answered	5
	
If treatment at our centre resulted in a successful twin delivery of two healthy babies (same sex twins) and you had extra embryos remaining in storage here, what do you think you would be most likely to do with them?	
Maintain them in storage, and plan for another attempt for pregnancy	97
Donate them for stem-cell research	6
Make them available for anonymous adoption by another couple	10
Undecided	87
Not answered	6
	
If treatment at our centre resulted in a successful twin delivery of two healthy babies (one boy + one girl) and you had extra embryos remaining in storage here, what do you think you would be most likely to do with them?	
Maintain them in storage, and plan for another attempt for pregnancy	90
Donate them for stem-cell research	6
Make them available for anonymous adoption by another couple	15
Undecided	89
Not answered	6

## Discussion

A global trend of delayed first pregnancy observed in developed countries translates into medical providers encountering the clinical problem of infertility more often [1]. This study offers a demographic snapshot of these patients as encountered in Ireland, along with clinical characteristics and reproductive history, as well as their expectations relative to certain treatment outcomes from IVF. It is the first attempt to quantify similarities or differences between husbands and wives regarding their fertility treatment expectations in Ireland.

It is perhaps not surprising that patients seeking a costly elective medical procedure that could include IVF would have done some research on the topic before the first reproductive endocrinology consultation. Indeed, we found that nearly all (94.7%) patients presenting for new IVF consult were already aware of links between fertility medications and multiple gestation. This observation could not be entirely explained by the fraction of patients (27.2%) with previous IVF experience, and probably reflects good counselling by other care providers (or self-instruction about infertility treatments) before arriving in a sub-specialty setting. However, our study did not quantify the depth of this patient knowledge nor the sources used to develop it.

It is important for IVF providers to explain statistics on treatment outcomes and associated risks in an understandable way, so that patient expectations are not set unrealistically. Likewise, it is critical for the physician to understand as much as possible about patients who commonly present for particular treatments. While negative emotions and stress must be recognised early, some anxiety is normal and the expression of negative emotions before IVF might not always impact outcomes negatively [2].

The current work offers insights into the level of concordance between husbands and wives who seek fertility treatment, regarding the disposition of surplus cryopreserved embryos that might derive from IVF. Additionally, we provide new data on how these patients perceive the possibility of having twins after IVF. These data show that there is good reported agreement between husbands and wives with respect to "readiness" for twins. We also found that 181 (91.3%) respondents had the same view on what to do with extra embryos, irrespective of whether the treatment eventually resulted in a twin delivery. There was a tendency for couples who are very excited about the prospect of having twins to be more likely to maintain their surplus embryos in frozen storage. We did not observe any evidence that patient age (either male or female) was associated with anxiety about twins in an upcoming fertility treatment cycle. Interestingly, ~10% of our new IVF patients in this sample expressed a qualified interest in either placing embryos in a stem cell research protocol or making them available to other couples anonymously via donation. Most (18/21) of these patients conditioned their preliminary bequest on a twin delivery from IVF, while three couples indicated they would still give their frozen embryos to stem cell research even if preceded by a singleton delivery. This observation, reported for the first time among Irish patients, echoes findings reported elsewhere where IVF patients designate embryo placement options that are not even available to them [3].

## Conclusion

Our previous research has confirmed that although the mean age of selected infertility patients in Ireland may have increased only nominally, there has been a sharp rise in the number of prior failed IVF cycles at baseline [4]. This means that fertility patients now likely represent a more refractory population and therefore bring a more guarded prognosis. Since no medical procedure is likely to overtake IVF as the leading form of assisted human reproduction [1], physicians encountering patients with an infertility diagnosis should have familiarity with this growing clinical group, as highlighted here.

## Competing interests

The authors declare that they have no competing interests.

## Authors' contributions

APHW was lead clinician, conceptualised the project and organised the research, GSC was chief statistician, DJW was consultant, MLD was principal nursing advisor and ESS was research consultant in reproductive medicine. All authors read and approved the final manuscript.
